# Epidemiology, disease evolution and economic burden of amyotrophic lateral sclerosis in France using the French national health data system

**DOI:** 10.1093/braincomms/fcaf292

**Published:** 2025-08-11

**Authors:** Philippe Corcia, Katie Stenson, Agathe Doutriaux, Hicham Hadjrabia, François Boer, Seham Issa, Sophie Marguet, Frederic Bernard, Enkhgerel Nasanbat, Gregoire Nowacki, Gérard de Pouvourville, Philippe Couratier

**Affiliations:** CRMR SLA, CHU de Tours, 37044 Tours, France; Global Value and Access, Biogen, Cambridge 02142, USA; Formerly of France Value and Access, Biogen, 92913 Paris La Défense, France; France Value and Access, Biogen, 92913 Paris La Défense, France; Formerly of France Medical Affairs Rare Diseases, Biogen, 92913 Paris La Défense, France; RWE Data and Analytics, Oracle Life Sciences, 75013 Paris, France; RWE Data and Analytics, Oracle Life Sciences, 75013 Paris, France; RWE Data and Analytics, Oracle Life Sciences, 75013 Paris, France; RWE Data and Analytics, Oracle Life Sciences, 75013 Paris, France; RWE Data and Analytics, Oracle Life Sciences, 75013 Paris, France; Department of Economics, ESSEC Business School, 95021 Cergy Pontoise, France; CRMR SLA, CHU de Limoges, 87000 Limoges, France

**Keywords:** amyotrophic lateral sclerosis, healthcare resource utilization, epidemiology, SNDS database, cost

## Abstract

Amyotrophic lateral sclerosis is a rare neurodegenerative disease that requires multidisciplinary care, resulting in extensive healthcare resource utilization. No study has explored the prevalence of amyotrophic lateral sclerosis or characterized associated healthcare resource utilization in France, despite its high burden on people living with amyotrophic lateral sclerosis, caregivers and the healthcare system. Herein, we conducted a France-wide retrospective study to describe the epidemiology, disease course and economic burden of amyotrophic lateral sclerosis. People living with amyotrophic lateral sclerosis were identified from the French population from 2012 to 2019 using the French national health data system. A milestone and symptom-based staging algorithm was developed to categorize the incident cohort into early, mid and late stages. Data were extracted on epidemiology, demographic and clinical characteristics and clinical treatments. Healthcare resource utilization and costs were analysed for amyotrophic lateral sclerosis cases and matched non-amyotrophic lateral sclerosis controls and at disease stages. Events of interests were identified, and a competing risk analysis was conducted. Comparative statistics were performed using paired *t*-test, ANOVA and χ^2^ test. We identified 18 289 incident amyotrophic lateral sclerosis cases, 56.1% of whom were male. The average age at diagnosis was 68.4 (± 12.5) years. In 2019, the estimated incidence and prevalence were 3.5/100 000 person-years and 11.0/100 000 persons, respectively. All-cause hospitalizations were higher among people at mid stage (1.66 person-years) and late stage (2.82 person-years) than among people at early stage (1.46 person-years) and for the amyotrophic lateral sclerosis cases (1.98 person-years) than for the matched non-amyotrophic lateral sclerosis controls (0.45 person-years). Home hospitalization and rehabilitation admission were more prevalent among people in later stages. The rate of out-patient and ambulatory consultations to all specialties was 13.8 person-years for people at early and mid stages and 11.1 person-years for people at late stage and 13.0 and 8.7 person-years for the amyotrophic lateral sclerosis cases and the matched non-amyotrophic lateral sclerosis controls, respectively. Direct costs increased as the disease progressed and were also higher for the amyotrophic lateral sclerosis cases than for the matched non-amyotrophic lateral sclerosis controls. Amyotrophic lateral sclerosis imposes a significant health burden through incremental healthcare resource utilization across all stages that increases with disease progression. Out-patient and ambulatory resource consumption decreased as the disease progressed to a more severe form, accompanied by a corresponding increase in in-patient services. These findings shed light on the complex needs of people living with amyotrophic lateral sclerosis and the continued need for more efficacious treatments.

## Teaser

Corcia *et al*. present real-world data on patients living with amyotrophic lateral sclerosis in France. Findings cover patient characteristics at onset, survival, time-to-event endpoints for artificial nutrition, ventilation, among other milestones, and healthcare resource use and associated costs per disease stage.

## Graphical Abstract

**Graphical Abstract:**
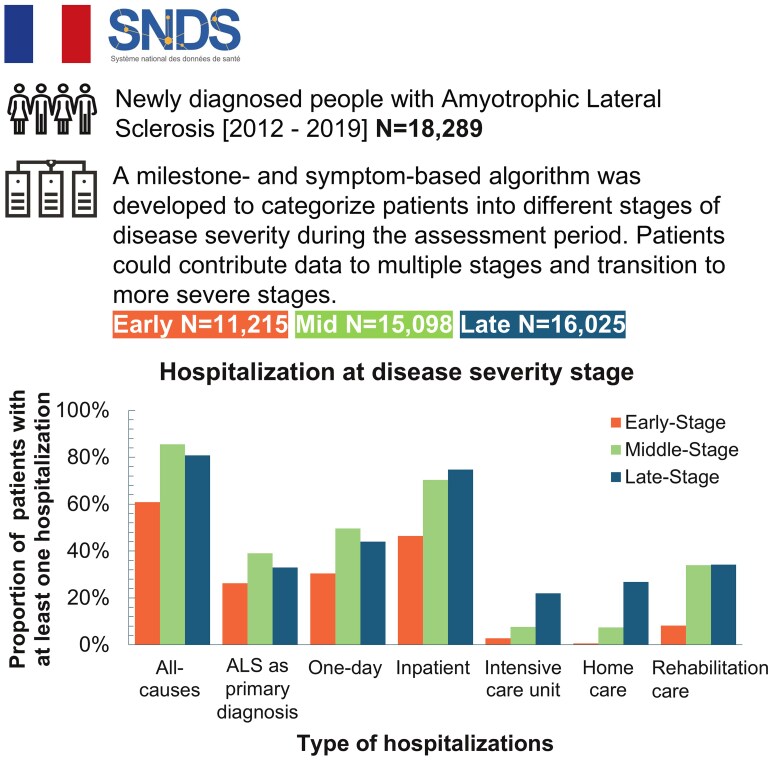

## Introduction

Amyotrophic lateral sclerosis (ALS) is a fatal neurodegenerative disorder characterized by progressive loss of the upper and lower motor neurons that involve the brain and spinal regions, resulting in motor disturbances and progressive paralysis.^1,2^ ALS is the most common type of motor neuron disease (MND) in adults, accounting for ∼90% of cases.^3^ With an onset age of 51–66 years,^4,5^ ALS symptoms can be broadly grouped into early-stage symptoms, such as fatigue, cramping, fasciculations, dysarthria, difficult swallowing, loss of motor skills and abnormal reflexes, middle-stage symptoms, such as sialorrhoea, falls, dysphagia, orthopnoea, dyspnoea, weight loss and moderate-to-severe malnutrition, and late-stage symptoms, such as paralysis of most voluntary muscles, respiratory failure and gastrostomy placement, that subsequently spread to different body parts and typically limit survival to 3–5 years from symptom onset.^6,7^ Staging criteria for ALS provide a structured framework to assess disease progression, which is essential for optimizing resource allocation and healthcare planning for people living with ALS (plwALS). While several clinical staging systems have been proposed for ALS, such as the King’s College clinical staging system for ALS and the ALS Milano-Torino staging (MiToS), these are usually unavailable in medico-administrative databases.^7-9^ To date, there is no universal definition to categorize patients into early, mid and late disease, and several efforts have been directed to propose staging criteria based on experts’ input.^6,10^

Given the heterogeneity in the clinical presentation and the incomplete understanding of ALS pathophysiology,^11^ early diagnosis remains an enormous challenge owing to symptoms that can mimic other MND types and the lack of reliable diagnostic biomarkers. Further, the probability of misdiagnosis is reported to be high and associated with a delay in treatment, which may have unfavourable effects on psychological or social health.^12^ When no alternative explanation can be found after exclusion of similar-presenting disorders using clinical, neurophysiologic, laboratory and neuroimaging findings, the progressive spread of motor symptoms serves as the basis of diagnosis.^13^

The clinical features of this debilitating disease increase the dependency of plwALS on caregivers and significantly affect their quality of life.^6,14^ The treatment of ALS requires multidisciplinary specialists such as neurologists, respiratory physicians, gastroenterologists, rehabilitation medicine physicians, social workers, occupational therapists, speech therapists, respiratory therapists, specialized nurses, physical therapists, dietitians, psychologists and palliative care physicians, with nutritional and respiratory support and symptom management, as the cornerstone of treatment.^15^ Riluzole, the only approved drug for broad ALS in France, is associated with survival gains of a few months.^3,16^

Despite the complexity of ALS as a multisystemic disease, studies investigating the global landscape of ALS economic burden are limited. Studies conducted to examine the economic burden of ALS reported the national costs to vary from €149 to €1329 million in Germany^17^ and 212 million to 1.4 billion USD/year in the USA.^18^ However, variations among countries in available treatments, government and insurance policies, and healthcare system structure, and other factors that drive ALS management limit the generalizability of these results. Further, the precise needs of plwALS in terms of medical consultations, hospitalization or out-patient care, medical aids, support from caregivers, etc., change throughout the course of the disease, and therefore, an accurate and thorough understanding of the real-world management and economic burden of ALS is needed. To this end, we performed a nationwide retrospective study from the public payer perspective to describe the epidemiology and disease evolution and analyse the economic burden of ALS at different stages and, in comparison with a matched random sample from the general population in France using the French national health data system, Système National des Données de Santé (SNDS).

## Materials and methods

### Study design and data source

A retrospective real-world study was conducted among a prevalent population of plwALS to investigate the epidemiological outcomes (prevalence, incidence and mortality) from 2012 to 2019 and among a longitudinal incident cohort identified during the same period to assess disease evolution, treatment use, health care resource use (HCRU) and costs. For plwALS included in the incident cohort, data for a minimum period of 2 years before the identified date of diagnosis (recorded diagnosis code or initiation of riluzole) were extracted, and a minimum of 1 year of follow-up after this date was required, except in case of death. The study period was, therefore, from 2010 to 2020.

The SNDS is a national administrative healthcare database in France that covers almost 99% of the French population from birth (or immigration) to death (or emigration).^19^ It links individual data for all healthcare expenditures reimbursed by the public mandatory French National Health Insurance, including out-patient care and hospitalization, and data from the national death registry.^20^ In particular, SNDS encompasses pseudonymized data related to sociodemographics (age, sex and region), physician and paramedical visits, medications and medical procedures, laboratory tests, hospital stays, date and costs of care and date of death.^20-22^ Diagnosis codes using the International Classification of Diseases, 10th Revision (ICD-10), can be identified in SNDS in hospital discharge summaries or by means of long-term chronic diseases specified in the list of diseases that are associated with complete reimbursement of healthcare-related costs by the main French health insurance system.^22,23^ SNDS is one of the largest medico-administrative databases with high completeness, quality and very low attrition rate.^24,25^ Data are recorded according to rigorous guidelines and coding systems. A standardized data quality plan is performed at several levels: during electronic data acquisition, data entry at processing centres and coding before integration into the national data warehouse.^20^

The study protocol was approved by a local scientific steering committee (SSC) consisting of two hospital-based neurologists specializing in treating plwALS and a health economist with expertise in real-world data. It was also reviewed by an independent committee (CESREES) and the French data protection agency [Commission Nationale Informatique et Libertés (CNIL)] that granted approval for data access. Data extraction from SNDS was regulated by a security process endorsement for data storage.^26^ The study followed the STROBE guidelines for reporting observational studies.^27^

### Study population

**Figure 1:**
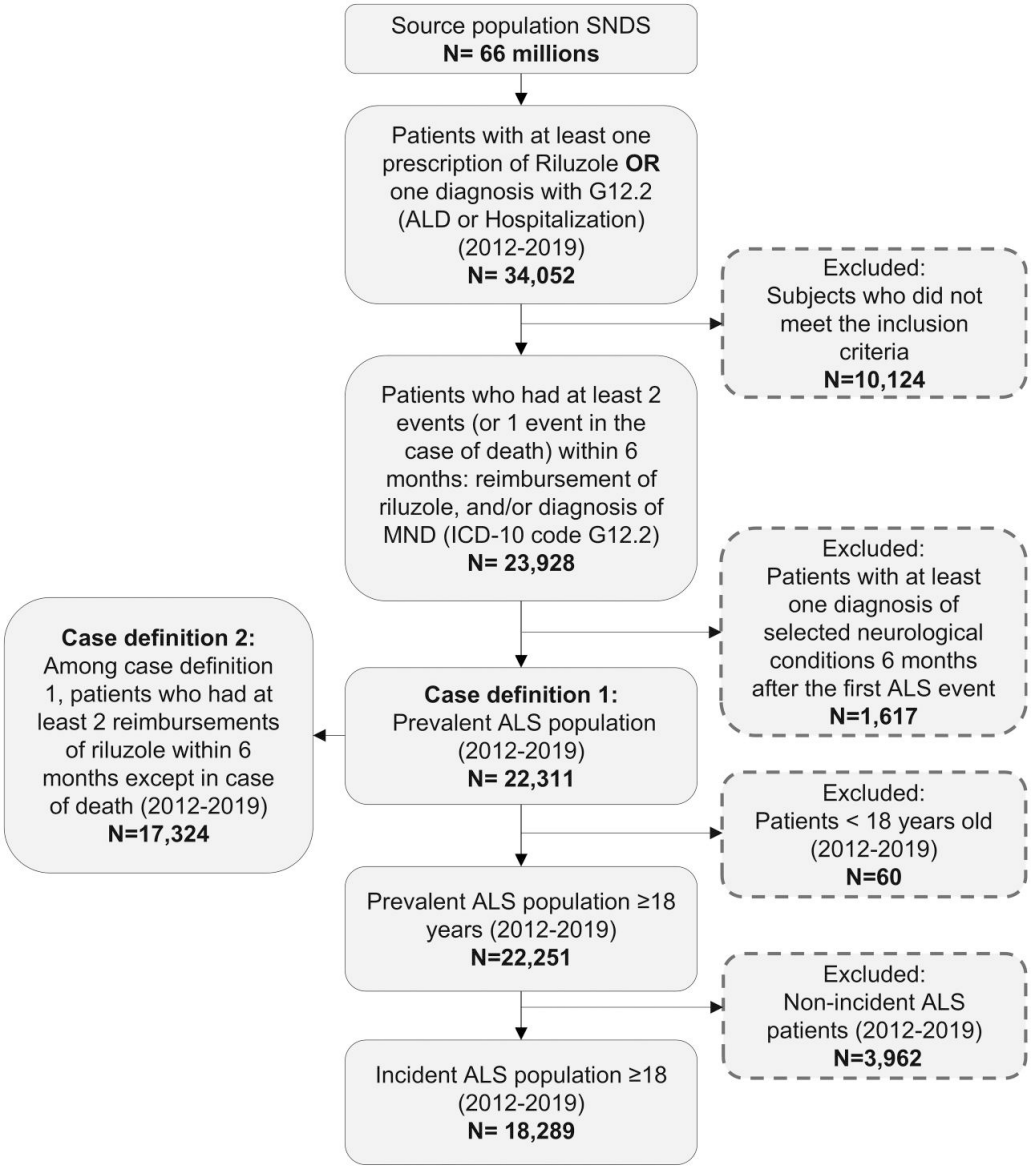
**Study population.** Flowchart of study population selection. ALD, Affection Longue Durée (long-term disease); MND, motor neuron disease.

plwALS were identified from the French population (around 66 million during the study period) using the SNDS database from 1 January 2012 to 31 December 2019 (Fig. 1). plwALS were defined as having at least two events (or one event in case of death) within a 6-month period as follows: a MND diagnosis based on the ICD-10 code G12.2 recorded during a hospitalization (all settings as a primary, linked or associated diagnosis) or as a long-term chronic disease and a reimbursement of riluzole, which is only approved for use in ALS. People were excluded if they had at least one diagnosis code occurring 6 months after the first ALS event for hereditary spastic paraplegia (G11.4), Parkinson’s disease (G20), multiple sclerosis (G35) and neurological conditions other than MND within G12.

From this prevalent population of people designated as having ALS, an incident cohort was built by including only newly diagnosed adult cases with no MND diagnosis or riluzole prescription during a period of 2 years prior to the identified date of diagnosis during the identification period (2012–19).

A milestone and symptom-based staging algorithm was developed by incorporating input from two local ALS physicians who were part of the SSC (Supplementary Table 1A). The final staging criteria were designed to be mutually exclusive between the different stages. The identified milestones and symptoms were then linked to their respective codes including diagnosis, medical procedures, medical devices, medication codes, among others (Supplementary Table 1B). The entire incident cohort was categorized into early, mid and late ALS stages based on these definitions. Stage onset was determined by the first occurrence of a milestone or symptom defining that stage (from 2 years before identified diagnosis until the end of follow-up), without the presence of events from more advanced stages. plwALS could contribute data to multiple stages and transition to more severe stages; however, disease progression to a later stage was considered irreversible. Follow-up could end due to death or end of study period.

In addition, a non-ALS control group was randomly selected from the general population that comprised individuals with no MND diagnosis or riluzole prescription between January 2010 and December 2020. All ALS cases from the incident cohort were matched with the non-ALS subjects from the random sample in a 1:3 ratio for year of birth, sex and region.

### Study variables and outcomes

#### Incidence, prevalence and mortality

The yearly prevalence of ALS was defined as the total number of plwALS each year from 2012 to 2019 (Case definition 1: as defined in study population), divided by the size of the French population for the same year. A conservative yearly prevalence was additionally estimated by restricting the number of plwALS to those who had at least two reimbursements of riluzole (or one in case of death) within a 6-month period (Case definition 2). The yearly incidence rate was determined as the number of people with a first documentation of ALS (recorded diagnosis code or initiation of riluzole) in SNDS each year, divided by the sum of the person-years (PY) at risk for the same year. Given that the number of patients living with ALS is relatively small compared with the general population, the total observation time accumulated by the at-risk population was approximated as the sum of PY of all individuals alive at the start of each calendar year. In addition, the yearly mortality rate was calculated as the number of all-cause deaths occurring in the ALS prevalent cases for each year, divided by the sum of PY of all individuals alive in that year. The denominators were derived from the French National Institute of Statistics and Economic Studies.^28^ The most common medical causes of death, as recorded in the national death registry, were reported from 2013 to 2017.

#### Demographics

Information was extracted from the database on plwALS’ demographics (age, sex, follow-up period, region of residence and social deprivation index) at initial diagnosis of ALS for the incident cohort.

#### Events of interest

To assess the disease evolution, 10 key events of interest were selected in consultation with the SSC, based on their clinical relevance as indicators of disease severity. These include indicators of moderate disease, such as short-term non-invasive ventilation (NIV) (<12 h) and reimbursement of manual wheelchair; severe disease indicators, including NIV (>12 h), invasive ventilation, reimbursement of electric wheelchair and admission to palliative care unit; and other milestone-related HCRU, such as artificial nutrition, overall wheelchair reimbursement, home hospitalization [Hospitalisation à domicile (HAD)] and rehabilitation care [Soins de Suite et de Réadaptation (SSR)]. Time-to-first event was defined as the time between the date of disease onset and the first record of the event. Disease onset was defined as the date of the first milestone or symptom (from Supplementary Table 1A) and could predate date of diagnosis. Overall survival from the date of disease onset was also assessed for the incident cohort.

#### Treatment use, HCRU and costs

ALS treatment with riluzole was described for the incident cohort. HCRU was assessed for the incident cohort in in-patient and out-patient settings and analysed at different disease stages and for ALS versus non-ALS population. All-cause and ALS-related hospitalization [1-day hospitalization, intensive care unit (ICU) admission, in-patient hospitalization], home hospitalization and rehabilitation care were identified and described. ALS-related hospitalization was defined as any hospitalization occurring in medical, surgical and obstetrics (MCO) with MND as the primary diagnosis. One-day hospitalization was defined when admission date is equal to discharge date, as opposed to in-patient hospitalizations lasting for at least 24 h. Resources based on out-patient and ambulatory visits were also documented. Consultations correspond to out-patient [Actes et consultations externes (ACE)] and ambulatory care. ACE does not cover 1-day hospitalizations. In France, plwALS can be seen during multidisciplinary consultations usually delivered in excellence centres. However, the granularity of SNDS does not allow to capture these types of encounters.

French healthcare insurance system consists of several schemes based on occupational sectors with broadly similar healthcare coverage for beneficiaries.^24^ In France, patients with long-term chronic diseases, including MND, are exempt from co-payments.^29^ Transportation costs for medical treatment are reimbursed with a healthcare provider’s prescription and are often fully covered for long-term chronic diseases. Sick leave benefits provide daily allowances, typically 50–66.6% of the employee’s average salary, and for long-term chronic illnesses, extended benefits and sick leave are available. In this study, direct costs reimbursed by the French public mandatory health insurance were estimated from the payer’s perspective and assessed by summing the actual fees associated with different settings. Costs of hospital stays in MCO were based on the diagnosis-related group (DRG) recorded in the SNDS and the corresponding national tariffs estimated in the national cost studies based on a common methodology led by the Technical Agency for Information on Hospital Care.^30^ Similarly, the costs of hospitalization at home and follow-up and rehabilitation care were calculated using the national tariffs from these studies^30^ and the recorded homogeneous healthcare group and medico-economic group (equivalent to DRG), respectively. Other medical costs (out-patient visits, selected paramedical care, medications and medical devices), sick leave costs (among the assumed active working age subset of 18–64 years) and transportation costs were directly extracted from SNDS based on the amount reimbursed by the public health insurance. Selected paramedical care included care provided by nurses, speech therapists, psycho-motor therapists or psychologists. Costs were provided per person-year in euros.

### Statistical analysis

All analyses were performed using SAS® 9.4 (SAS Institute, Inc., Cary, NC, USA). Descriptive analyses were performed using standard descriptive statistics. Continuous variables were presented as mean (standard deviation) and median (interquartile range), and categorical variables were expressed as frequencies and percentages. For categorical variables, missing observations, if any, were considered as a separate category. For continuous variables, between-group comparisons were made using a Student’s *t*-test or ANOVA in case of more than two groups. For categorical variables, χ^2^ test was used for comparison. HCRU and costs were analysed using PY to account for varying follow-up times of patients, ensuring each individual’s contribution to the analysis is appropriately adjusted regardless of their specific follow-up duration. Overall survival analysis was conducted using the Kaplan–Meier method, and the results were expressed as median time [95% confidence interval (CI)]. For the events of interest, a competing risk analysis was conducted with death as a competing event. The results were presented as cumulative incidence at 1, 3 and 5 years (95% CI).

## Results

### Study population

Figure 1 depicts the flowchart of the selection steps employed to identify the ALS population in this study. Between 2012 and 2019, we identified from the SNDS database 23 928 people with at least two events (or one event in case of death): reimbursement of riluzole or diagnosis code for MND within 6 months. After applying exclusion criteria, the plwALS prevalent population included 22 251 and 17 324 per Case definitions 1 and 2, respectively, and 18 289 plwALS as the incident cohort in the analysis.

We used the staging algorithm shown in Supplementary Table 1A to categorize incident plwALS depending on their ALS disease stage evolution during the follow-up period into early (11 215), mid (15 098) and late (16 025) stages. In addition, we employed a matched non-ALS population comprising 54 867 subjects for comparative analyses.

### Baseline demographics

The 18 289 eligible incident plwALS comprised 10 252 (56.1%) men and 8037 (43.9%) women (Table 1). The mean (SD) age at diagnosis was 68.4 (12.5) years, and the mean duration of follow-up from diagnosis was 760.0 (SD 754.4) days. Overall, 6369 (34.8%) and 648 (3.5%) plwALS were living in the most deprived geographic areas (Q4 and Q5, respectively). In terms of region of residence, plwALS were distributed across all regions including overseas territories, with most cases in metropolitan area. Aside from the similarities in the characteristics included in the matching (age, sex and region), the patients in the ALS group were similar to those in the non-ALS group in terms of social deprivation index and receipt of state aids (data not shown).

**Table 1:** Baseline demographic characteristics of the incident ALS population

	ALS population *n* = 18 289
**Age at index date**	
Mean (SD)	68.35 (12.50)
Median	69
Q1–Q3	61–78
Min, max	18–100
**Gender**	
Male	10 252 (56.06%)
Female	8037 (43.94%)
**Follow-up period (days)**	
Mean (SD)	760.02 (754.39)
Median	516
Q1–Q3	201–1038
**Social deprivation index (2009), *n* (%)**	
Q1 high—more advantaged geographic area	1677 (9.17%)
Q2	4176 (22.83%)
Q3	5168 (28.26%)
Q4	6369 (34.82%)
Q5 low—more deprived geographic area	648 (3.54%)
Missing	251 (1.37%)
**Region or department of residence, *n* (%)**	
Missing	3 (0.02%)
Auvergne-Rhône-Alpes	2279 (12.46%)
Bourgogne-Franche-Comte	777 (4.25%)
Bretagne	1095 (5.99%)
Centre-Val De Loire	773 (4.23%)
Corse	73 (0.4%)
Grand Est	1419 (7.76%)
Hauts-De-France	1452 (7.94%)
Ile-De-France	2944 (16.1%)
Normandie	988 (5.4%)
Nouvelle-Aquitaine	1762 (9.63%)
Occitanie	1799 (9.84%)
Pays De La Loire	1144 (6.26%)
Provence-Alpes-Côte D'Azur	1533 (8.38%)
Overseas territories^a^	248 (1.36%)

^a^Overseas territories include Guadeloupe, La Réunion, Guyane, Martinique, Mayotte, and unspecified overseas territories.

### Prevalence, incidence and mortality

**Figure 2:**
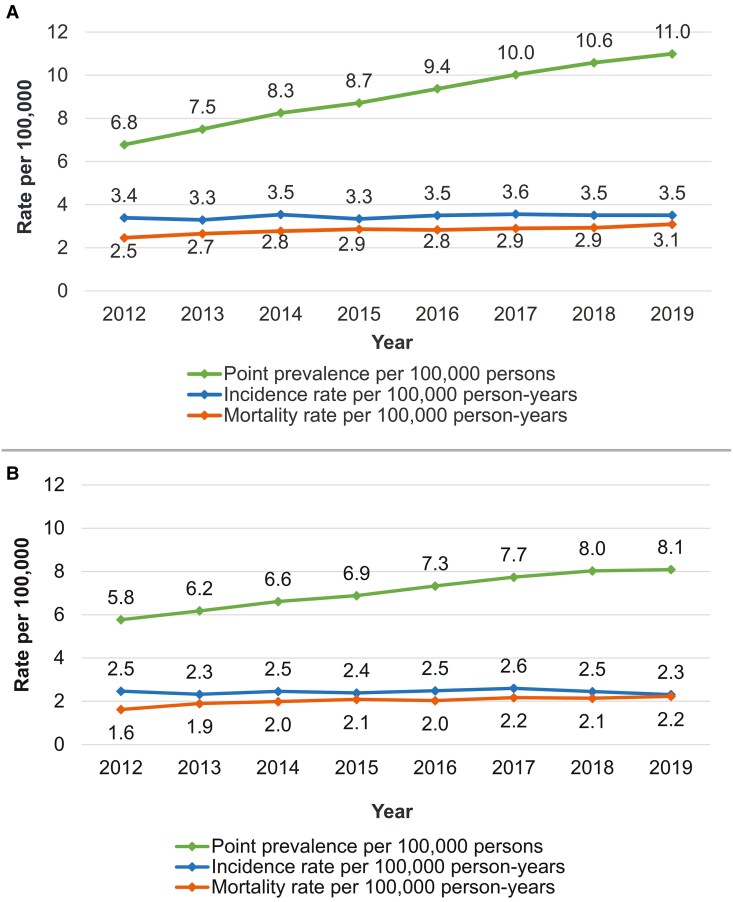
**Prevalence, incidence and mortality rate of ALS in France.** (**A**) Estimated crude prevalence, incidence and mortality of ALS, 2012–19, using Case definition 1 (*n* = 22 311 in total) and annual estimates of the French population (National Institute of Statistics and Economic Studies). (**B**) Estimated crude prevalence, incidence and mortality of ALS, 2012–19, using Case definition 2 (*n* = 17 324 in total) and annual estimates of the French population (National Institute of Statistics and Economic Studies).

From 2012 to 2019, the point prevalence of ALS increased from 6.8 to 11.0 cases per 100 000 persons using Case definition 1 (Fig. 2A). During the same period, the estimated incidence of ALS remained stable between 3.4 and 3.5 cases per 100 000 PY (Fig. 2A). The mortality rate varied from 2.5 to 3.1 cases per 100 000 PY from 2012 to 2019 (Fig. 2A).

Considering an alternative case definition with riluzole users only (Case definition 2), the ALS prevalence rose from 5.8 cases per 100 000 persons in 2012 to 8.1 cases per 100 000 persons in 2019 (Fig. 2B). The incidence of ALS among riluzole-treated people remained relatively the same from 2012 to 2019, with the highest rate being reported in 2017, i.e. 2.6 cases per 100 000 PY (Fig. 2B). The mortality rate slightly rose from 1.6 deaths per 100 000 PY in 2012 to 2.2 deaths per 100 000 PY in 2019 (Fig. 2B). The most common causes of death among plwALS analysed between 2013 and 2017 were respiratory complications, including arrest (29.0–25.4%), acute respiratory failure (23.9–20.4%) and unspecified respiratory failure (9.4–8.2%) (Supplementary Table 2).

### Disease evolution

Table 2 describes the time to events of interest from the ALS disease onset. Of 18 289 included incident plwALS, 4952 (27.1%) had received artificial nutrition during the follow-up. The cumulative incidence of artificial nutrition over the first year was 0.10 (95% CI, 0.10–0.11), which rose to 0.25 (95% CI, 0.24–0.25) over 3 years and 0.28 (95% CI, 0.27–0.28) over 5 years. During the follow-up, 7214 (39.4%) and 2597 (14.2%) plwALS were supported by <12- and >12-h NIV, respectively. The cumulative incidences of <12 h and >12 h NIV over 1, 3 and 5 years were 0.16 (95% CI, 0.15–0.17), 0.35 (95% CI, 0.35–0.36) and 0.40 (95% CI, 0.39–0.41) and 0.04 (95% CI, 0.04–0.04), 0.12 (95% CI, 0.11–0.12) and 0.14 (95% CI, 0.14–0.15), respectively. In addition, 1364 (7.5%) plwALS were reported to have received invasive ventilation support; the cumulative incidence of invasive ventilation was 0.04 (95% CI, 0.04–0.04) over the first year and increased to 0.12 (95% CI, 0.11–0.12) over 3 years and 0.14 (95% CI, 0.14–0.15) over 5 years.

**Table 2:** Events of interest

	ALS population *n* = 18 289				
Event of interest	Yes, *n* (%)	No, *n* (%)	Cumulative Incidence over 1 year (SD)	Cumulative Incidence over 3 years (SD)	Cumulative Incidence over 5 years (SD)
Artificial nutrition^a^	4952 (27.08%)	13 337 (72.92%)	0.10 (0.10–0.11)	0.25 (0.24–0.25)	0.28 (0.27–0.28)
Non-invasive ventilation <12h^b^	7214 (39.44%)	11 075 (60.56%)	0.16 (0.15–0.17)	0.35 (0.35–0.36)	0.40 (0.39–0.41)
Non-invasive ventilation >12h^b^	2597 (14.20%)	15 692 (85.80%)	0.04 (0.04–0.04)	0.12 (0.11–0.12)	0.14 (0.14–0.15)
Invasive ventilation^c^	1364 (7.46%)	16 925 (92.54%)	0.04 (0.04–0.04)	0.12 (0.11–0.12)	0.14 (0.14–0.15)
Reimbursement of wheelchair	10 452 (57.15%)	7837 (42.85%)	0.27 (0.27–0.28)	0.54 (0.53–0.54)	0.58 (0.57–0.59)
Reimbursement of manual wheelchair	9247 (50.56%)	9042 (49.44%)	0.03 (0.03–0.03)	0.24 (0.23–0.24	0.47 (0.47–0.48)
Reimbursement of electric wheelchair	5198 (28.42%)	13 091 (71.58%)	0.09 (0.08–0.09)	0.25 (0.24–0.26)	0.29 (0.29–0.3)
Home care (HAD)	3653 (19.97%)	14 636 (80.03%)	0.06 (0.06–0.06)	0.17 (0.17–0.18)	0.2 (0.2–0.21)
Rehabilitation care (SSR)	6852 (37.47%)	11 437 (62.53%)	0.19 (0.19–0.2)	0.35 (0.35–0.36)	0.38 (0.37–0.39)
Admission to palliative care unit	6740 (36.85%)	11 549 (63.15%)	0.12 (0.12–0.13)	0.32 (0.32–0.33)	0.38 (0.37–0.38)

^a^Artificial nutrition is defined as the first administration of enteral or parenteral nutrition.

^b^Non-invasive ventilation is a type of ventilation not requiring tracheal intubation.

^c^Invasive ventilation is a type of ventilation that involves an endotracheal tube or tracheostomy tube to create an airway placed inside the trachea.

Overall, 10 452 (57.2%) plwALS were reimbursed for wheelchair during the follow-up, and the cumulative incidence of this reimbursement was 0.27 (95% CI, 0.27–0.28) over 1 year, increasing to 0.54 (95% CI, 0.53–0.54) over 3 years and 0.58 (95% CI, 0.57–0.59) over 5 years. Moreover, 9247 (50.6%) plwALS were reimbursed for manual wheelchairs and 5198 (28.4%) for electric wheelchairs. The cumulative incidence of reimbursement for manual wheelchairs was 0.03 (95% CI, 0.03–0.03) over 1 year, 0.24 (95% CI, 0.23–0.24) over 3 years and 0.47 (95% CI, 0.47–0.48) over 5 years. For electric wheelchairs, the cumulative incidence was 0.09 (95% CI, 0.08–0.09) over 1 year, 0.25 (95% CI, 0.24–0.26) over 3 years and 0.29 (95% CI, 0.29–0.3) over 5 years. In total, 3653 (20.0%) and 6852 (37.5%) plwALS had at least one HAD and one admission to SSR, respectively. The cumulative incidence over 1, 3 and 5 years of HAD was 0.06 (95% CI, 0.06–0.06), 0.17 (95% CI, 0.17–0.18) and 0.2 (95% CI, 0.2–0.21), respectively, and of SSR admission was 0.19 (95% CI, 0.19–0.2), 0.35 (95% CI, 0.35–0.36) and 0.38 (95% CI, 0.37–0.39), respectively. In total, 6740 (36.9%) plwALS were admitted to a palliative care unit; the cumulative incidence of palliative care admission increased from 0.12 (95% CI, 0.12–0.13) over the first year to 0.32 (95% CI, 0.32–0.33) over 3 years and 0.38 (95% CI, 0.37–0.38) over 5 years.

**Figure 3:**
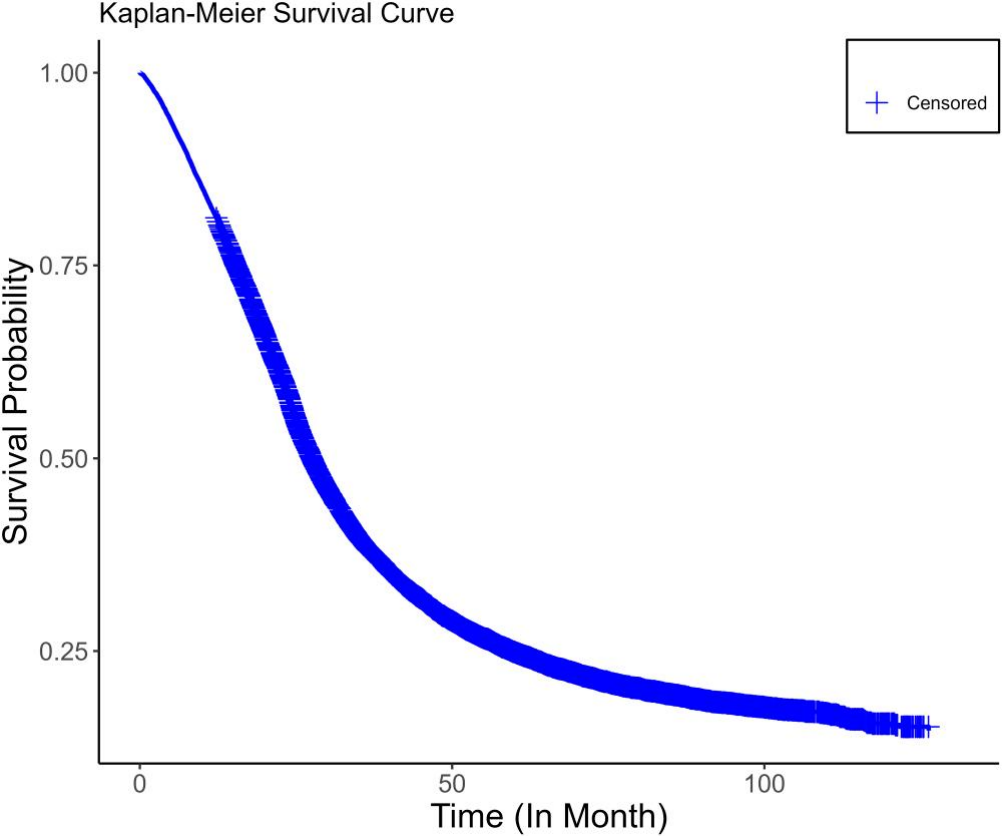
**Kaplan–Meier curve displaying the overall survival from the date of ALS onset.** Incident people with ALS (2012–19) *n* = 18 289. *Statistical method:* The Kaplan–Meier survival curve illustrates the overall survival of the entire incident people with ALS cohort. Overall survival is measured in months and defined as the time from the first recorded ALS event to death from any cause. The percentage of patients at risk at various follow-up time points is displayed with the end of study period as a censoring event.

The median (95% CI) overall survival among plwALS from the time of ALS onset was 27.6 (27.2–27.9) months (Fig. 3).

### Treatment use

Most of the incident plwALS (13 455 of 18 289; 73.6%) were ever treated with riluzole during the follow-up (Supplementary Table 3). For these plwALS, the mean time from identified ALS diagnosis to riluzole treatment initiation was less than a month [0.66 months (SD 1.7)]. The mean duration of treatment with riluzole was 18.6 months (SD 18.1).

### HCRU and related cost at disease stage

#### Hospitalization at disease stage

**Figure 4:**
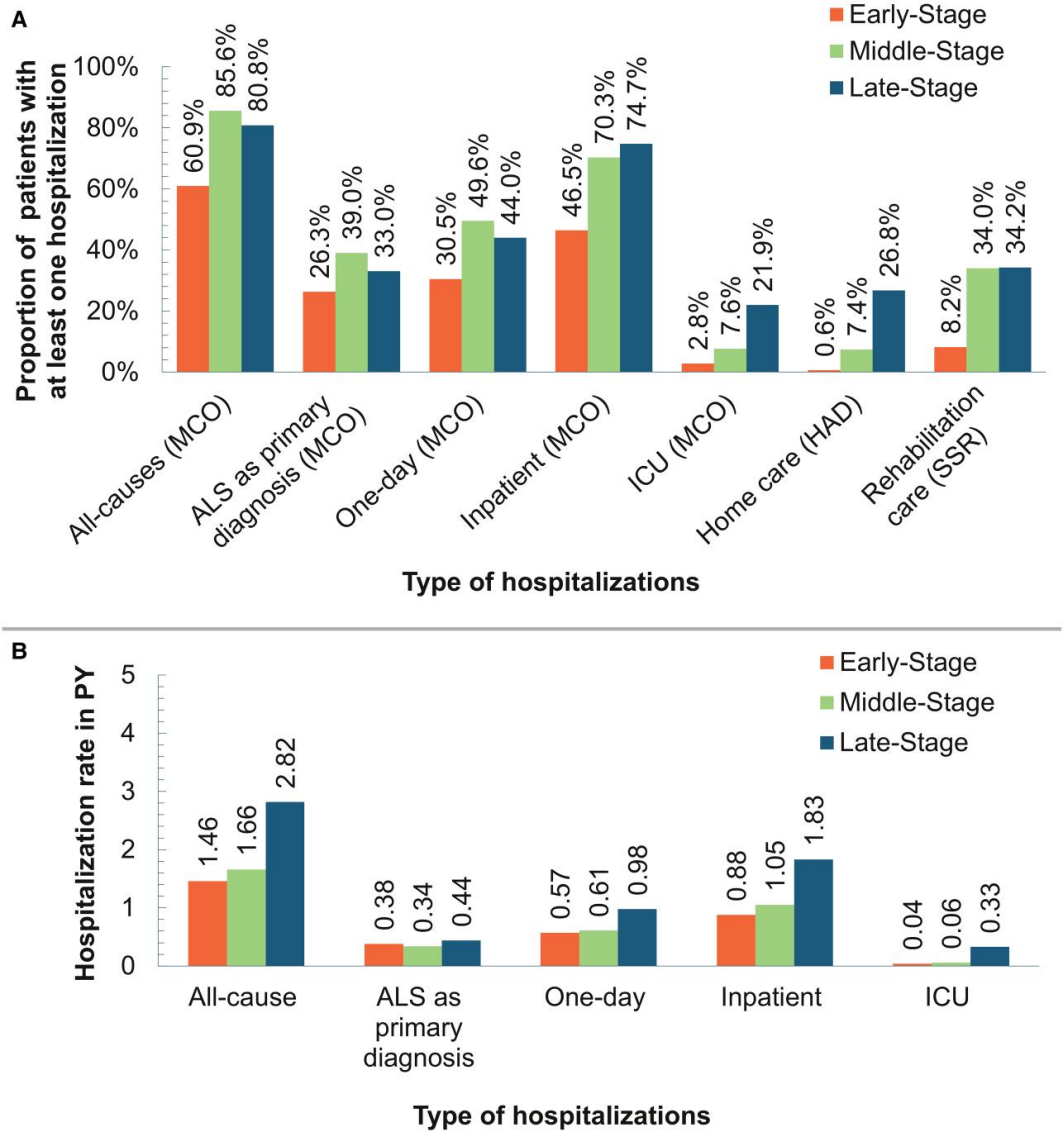
**Hospitalization among plwALS at disease severity stage.** (**A**) Proportion of patients with at least one hospitalization (MCO/HAD/SSR) at disease severity stage. Early stage *n* = 11 215; mid stage *n* = 15 098; late stage *n* = 16 025. *Statistical method*: χ^2^ test was used, and all observed differences were statistically significant (*P* < 0.01). (**B**) Hospitalization rate in patient-years (MCO) at disease severity stage. Early stage *n* = 11 215; mid stage *n* = 15 098; late stage *n* = 16 025. *Statistical method*: ANOVA was used, and all observed differences were statistically significant (*P* < 0.01). MCO, medical, surgical and obstetrics; HAD, home care; SSR, rehabilitation care; ICU, intensive care unit; PY, person-year.

As shown in Fig. 4A, the proportion of incident plwALS having at least one all-cause hospitalization or hospitalization with ALS as a primary diagnosis increased as the disease advanced from early (60.9 and 26.3%, respectively) to middle stage (85.6 and 39.0%) and decreased from middle to late stage (80.8 and 33.0%) (*P* < 0.01). The proportion of incident plwALS experiencing at least one in-patient hospitalization and ICU admission increased as the disease progressed from early (46.5 and 2.8%, respectively) to mid (70.3 and 7.6%) to late (74.7 and 21.9%) stage (*P* < 0.01). The all-cause hospitalization rate increased from 1.46 to 1.66 to 2.82 PY with the progression of the disease from the early to mid to late stage, respectively (*P* < 0.01; Fig. 4B).

Incident plwALS with advanced disease were more likely to have a home hospitalization or an admission to a rehabilitation centre, with higher proportions of plwALS with at least one event in late (26.8 and 34.2%, respectively) and mid (7.4 and 34.0%, respectively) stages than in the early stage (0.6 and 8.2%, respectively) (*P* < 0.01).

#### Consultations at disease stage

The percentages of incident plwALS with at least one out-patient and ambulatory consultation to physicians (all specialties), general practitioners (GPs) and neurologists remained higher among the plwALS with early- and mid-stage disease than among plwALS with late-stage disease (Supplementary Fig. 1A). More than 93% of plwALS had at least one consultation to a physician during the early and mid stages, compared with 67.3% in the late stage (*P* < 0.01). Overall, the rate of out-patient and ambulatory consultation to physicians of all specialties was 13.8 PY in the early- and mid-stage plwALS and 11.1 PY in the late-stage plwALS (*P* < 0.01; Supplementary Fig. 1B). Consultations to GPs varied from 7.9 PY among the early-stage group to 9.1 PY among the mid-stage group and 8.1 PY among the late-stage group (*P* < 0.01).

#### Costs at disease stage

The total direct costs comprised the estimated expenditures on hospital admission to MCO, HAD and SSR, consultations to physicians, medications delivered at the pharmacy, paramedical care, medical devices, transportation and sick leaves in the incident 18 289 plwALS (Table 3).

**Table 3:** Costs by disease stage and for ALS and non-ALS patient populations

		Per patient per year (€)					
Costs	ALS population	Non-ALS population ^f^	*P*-value^g^	ALS Early stage	ALS Middle stage	ALS Late stage	*P*-value
*n*	18 288	54 867		11 214	15 097	16 025	
Total direct costs ^a^	17 887	4174	<0.01	8240	13 752	28 307	<0.01
Total direct medical costs ^b^	16 947	4029	<0.01	7473	12 842	27 212	<0.01
Hospitalizations costs (MCO)	6763	1551	<0.01	3413	4795	11 017	<0.01
Medical imaging costs	156	136	<0.01	252	175	77	<0.01
Laboratory tests costs	556	441	<0.01	649	558	492	<0.01
ICU admission costs	1081	334	<0.01	226	376	2396	<0.01
Medication costs ^c^ (In-patient)	737	301	0.02	1025	861	379	<0.01
Consultations costs (Out-patient and ambulatory)	698	220	<0.01	595	709	737	<0.01
Home care admission costs (HAD)	1254	28	<0.01	77	456	2937	<0.01
Rehabilitation admission costs (SSR)	1281	190	<0.01	381	1028	2089	<0.01
Medication costs (Out-patient)	1561	654	<0.01	1258	1550	1732	<0.01
Paramedical care costs	2118	315	<0.01	868	1879	3087	<0.01
Medical device costs	3273	1070	<0.01	881	2426	5613	<0.01
Total direct non-medical costs ^d^	940	145	<0.01	768	910	1095	<0.01
Transport costs	328	41	<0.01	76	254	556	<0.01
Total sick leave payment ^e^	612	104	<0.01	691	656	539	<0.01

^a^The total direct costs include total direct medical costs and total direct non-medical costs.

^b^Total direct medical costs include hospitalizations (MCO, HAD, SSR), consultations with physicians, medications delivered at the pharmacy, paramedical care, medical devices.

^c^Medications from ‘liste-en-sus’ including medications dispensed by hospital pharmacies and expensive medications billed by the hospital.

^d^Total direct non-medical costs include transportation, and sick leaves.

^e^The costs related to sick leaves are available for the general scheme excluding local mutualist sections.

^f^The non-ALS population is representative of the overall non-ALS population in France with same age, sex and region as the ALS population in our study (matching). For information, in this non-ALS population, the proportion of people having a recorded diagnosis of hypertension and Type 2 diabetes (without complications) during the pre-index period was 24 and 5%, respectively.

^g^Between-group comparisons were made using the Student *t*-test or ANOVA, in the case of more than two groups.

The total direct (medical and non-medical) costs were 8240€/PY for the early-stage plwALS and increased to 13 752€/PY for the mid-stage plwALS and 28 307€/PY for the late-stage plwALS (*P* < 0.01).

The total direct medical costs also varied from 7473€/PY for early-stage plwALS to 12 842€/PY for mid-stage plwALS and 27 212€/PY for late-stage plwALS (*P* < 0.01).

The total direct non-medical costs, including transportation and sick leave reimbursement, were 768€/PY, 910€/PY and 1095€/PY for early-, mid- and late-stage plwALS, respectively (*P* < 0.01). We also estimated sick leave costs for the assumed active working age subset (18 to 64 years) of 6254 plwALS. The costs were 1631€/PY for 4079 early-stage plwALS, 1593€/PY for 5241 mid-stage plwALS and 1211€/PY for 5130 late-stage plwALS (*P* < 0.01).

### HCRU and related cost among ALS versus non-ALS population

#### Hospitalization for ALS versus non-ALS population

**Figure 5:**
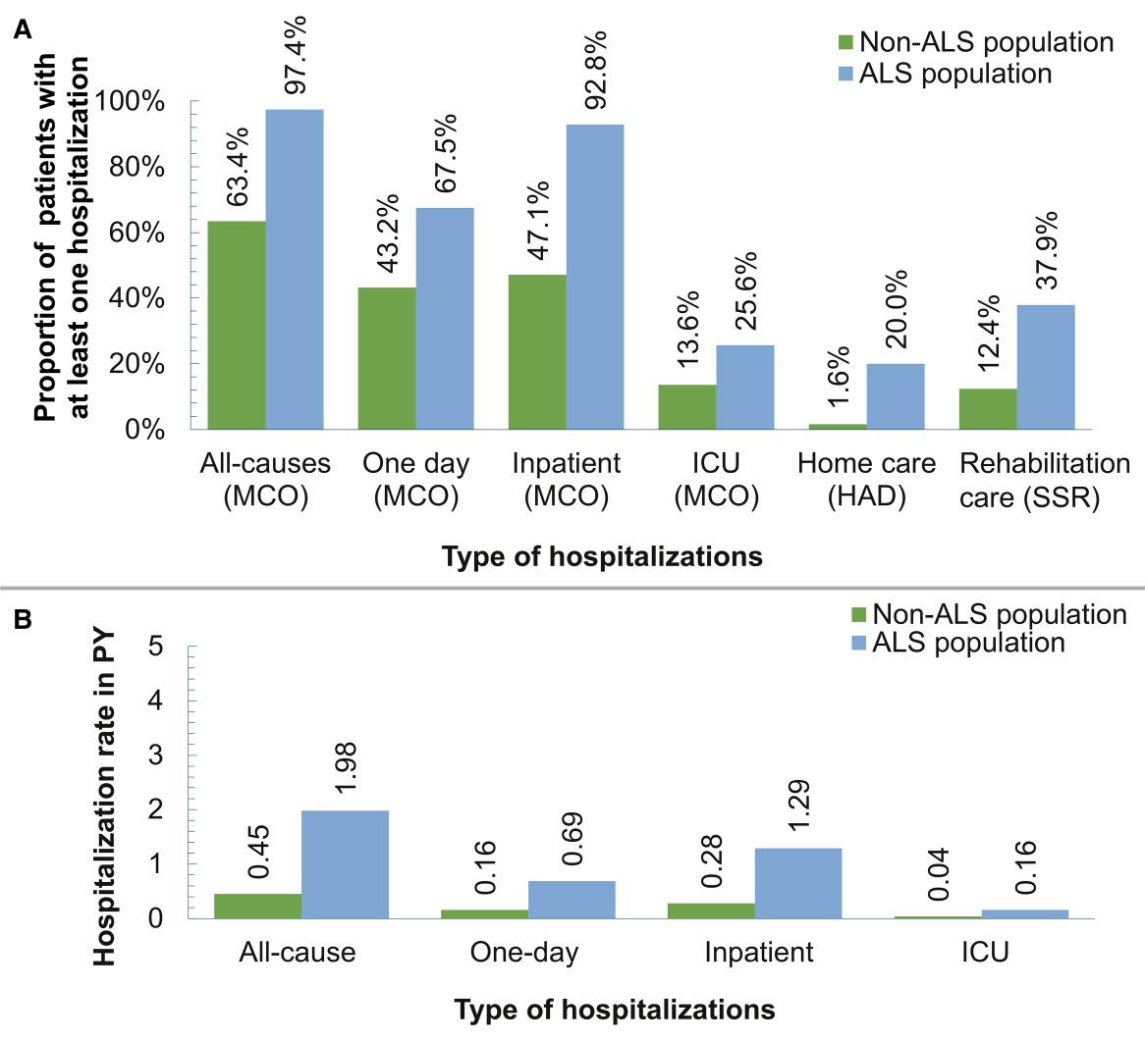
**Hospitalization among ALS and non-ALS group.** (**A**) Proportion of patients with at least one hospitalization (MCO/HAD/SSR) for ALS versus non-ALS population. Non-ALS population *n* = 54 867; ALS population *n* = 18 289. *Statistical method*: χ^2^ test was used, and all observed differences were statistically significant (*P* < 0.01). (**B**) Hospitalization (MCO) rates in patient-years for ALS versus non-ALS population. Non-ALS population *n* = 54 867; ALS population *n* = 18 289. *Statistical method*: Student’s *t*-test was used, and all observed differences were statistically significant (*P* < 0.01). MCO, medical, surgical and obstetrics; HAD, home care; SSR, rehabilitation care; ICU, intensive care unit; and PY, person-year.

As shown in Fig. 5A, the proportions of incident plwALS with at least one hospitalization (MCO, HAD and SSR) were higher among the ALS group (97.4, 20.0 and 37.9%, respectively) than among the non-ALS group (63.4, 1.6 and 12.4%, respectively) (*P* < 0.01). The proportion with at least one ICU and in-patient hospitalization were also higher among the ALS population (25.6 and 92.8%, respectively) than among the non-ALS control population (13.6 and 47.1%, respectively) (*P* < 0.01). The all-cause hospitalization rate was 1.98 PY among the ALS population, compared with 0.45 PY among the non-ALS population (*P* < 0.01; Fig. 5B).

#### Out-patient consultations for ALS versus non-ALS population

Out-patient consultations to physicians, GPs, neurologists and pneumologists were higher among the incident ALS population than among the non-ALS population (*P* < 0.01; Supplementary Fig. 2A). The rate of out-patient consultation to physicians of all specialties was 13.0 and 8.7 PY for the incident ALS and non-ALS population, respectively (*P* < 0.01; Supplementary Fig. 2B).

#### Cost among ALS and non-ALS group

The total direct (medical and non-medical) cost among all incident plwALS was 17 887€/PY, which was higher than that for the non-ALS group (4174€/PY) (*P* < 0.01). Similar trends were observed in both direct medical and non-medical costs, which were higher for the incident ALS population (16 947 and 940€/PY, respectively) compared with the non-ALS control population (4029 and 145€/PY, respectively) (*P* < 0.01; Table 3). The sick leave costs among the 6254 assumed active working age incident plwALS subset and 6227 non-ALS controls were 1444€/PY and 168€/PY (*P* < 0.01), respectively.

## Discussion

Using the French hospital administrative data (SNDS database), we identified plwALS between 2012 and 2019 to explore the epidemiology, disease course and economic burden of ALS in France. In Europe, various studies have reported the annual incidence of ALS ranging from 1.75 to 3.8 and the annual prevalence ranging from 4.1 to 12 cases per 100 000 individuals, with significant differences observed between geographical areas.^4,31-33^ In the present study, the annual incidence of ALS remained largely stable during the study period and ranged from 3.3 to 3.6 cases per 100 000 PY, which is in line with these previous reports as well as the temporal pattern reported in Italy^34^ where the mean annual crude incidence was 2.90 per 100 000 population (95% CI, 2.72–3.09) in 1995–2004 and did not fluctuate during the 10-year study period and in Limousin (former region in France) from the FRALim registry^35^ where the reported crude incidence was 3.26 per 100 000 PY of follow-up (95% CI, 2.97–3.55) and remained relatively stable, while clinical variability was observed. To the best of our knowledge, this is the first study to report the point prevalence of ALS in France that rose from 6.8 to 11.0 cases per 100 000 persons over the study period. The crude mortality rate remained largely static and consistently lower than the incidence rates during this period, which may potentially explain the steady rise in the crude prevalence of ALS observed herein.

The comprehensive analysis of the demographic and clinical characteristics shows that our ALS population have comparable attributes relative to those previously described.^36^ The disease tended to be slightly more common among men than among women (56.1 versus 43.9%). Further, the mean age of ALS onset was 68.4 years, consistent with that reported in Limousin (69 years)^35^ and Italy (64.8 years).^34^ In our study, the median overall survival from the time of the onset of ALS was 27.6 months, which aligns with the median overall survival from symptom onset of 30.5 months reported in the Piemonte and Valle d’Aosta ALS Register.^37^

The majority of the plwALS (73.6%) initiated treatment with riluzole, similar to usage reported in the previous SNDS-based study that identified incident MND cases in France between 2012 and 2014^3^ and other studies in Scotland^38^ and the USA.^6^ The initiation of riluzole treatment closely followed diagnosis (0.66 months), consistent with the local guidelines recommending prompt riluzole treatment for ALS management; however, considering that it is the only approved ALS treatment in France, one might expect most if not all plwALS to have at least one recorded usage of riluzole over the course of their disease.

The utilization of healthcare resources, in terms of healthcare professional (HCP) consultations, mobility aids, ventilation devices and caregiver assistance, differed between plwALS from the three severity groups, which underscores the substantial HCRU and financial burden through all stages of ALS and its escalation as the disease progresses to more severe stages as previously observed in other countries.^6^ For all-cause hospitalizations and those where ALS was the primary diagnosis, the proportion of plwALS having at least one event increased as the disease advances from early to mid stage and decreased from mid to late stage. The proportion of plwALS experiencing at least one in-patient hospitalization and admission to ICU increased significantly as the disease progressed to the late stage. The percentage of plwALS with at least one consultation with physicians (all specialties) remained high in the early and mid stages, decreasing slightly in the late stage. Underutilization of out-patient and ambulatory care in late-stage ALS (including consultations with neurologists) was seen along with an increase of hospitalization rates in MCO and in the proportion of plwALS having at least one admission in home care and rehabilitation as the disease progresses. These findings highlight the importance of alternative care settings for later-stage patients who might prefer to stay at home or have in-patient hospital stays due to mobility challenges or those requiring non-invasive/invasive ventilation.

Our analysis showed that in comparison with the non-ALS control group, the ALS patient population had substantially higher all-cause hospitalization, ICU admissions and 1-day and in-patient hospitalization. Several factors, particularly respiratory failure and need for invasive respiratory care, may have contributed to the higher burden on plwALS.^39^ Further, there were disparities in HCRU between the two groups. plwALS were significantly more likely to have consultations and had significantly higher visit frequencies to all specialties, highlighting their greater need for specialized and multidisciplinary medical attention. Interestingly, GPs seemed to play a more prominent role in ALS management than neurologists who are recommended to be seen quarterly (8.6/PY versus 0.9/PY).^40,41^ This observation is in line with previous results from a French SNDS study^42^ and highlights the important role of primary care providers in the comprehensive management of ALS in France but also may be impacted by the breakdown of physicians seen during multidisciplinary visits not being comprehensively captured in SNDS. Notably, in terms of national density in France, GPs and neurologists differ drastically, with 146.6 and 4.56 professional per 100 000 French residents, respectively, as of January 2023.^43^

The cumulative incidences of all the events of interest analysed herein increased with time, which underscores the loss of productivity and the inevitable decline in quality of life of plwALS as the disease progresses.^10,44^ This also supports that people with advanced disease may have great nursing requirements and need additional support beyond that provided by family members.^45^ The proportion of patients on invasive ventilation in our study was similar to that (9.5%) reported among 2702 ALS patients in Germany.^46^ A considerable proportion of the patients (36.9%) in our study received palliative care, consistent with the reported value of 42% by Salzmann *et al*.^47^ in Germany. Together, these observations reiterate the findings of the real-world study conducted using the Adelphi ALS Disease Specific Programme^TM^ in France, Germany, Italy, Spain, the UK and the USA where the proportion of patients who were completely care-dependent significantly increased from early to mid and late stages of ALS.^6^

We observed relatively high utilization of wheelchair among our cohort, as evident from reimbursements received by more than half of the plwALS. Noteworthy, SNDS only gathers information on wheelchair expenditures that are formally reimbursed and may not effectively capture the actual resource usage. The percentage of plwALS on invasive ventilation (7.5%) observed in our study is consistent with that reported in other countries.^48^ The use of NIV (14.2%) is in line with the results of a Canadian study where only 18.3% plwALS used NIV.^48^ In Europe and the USA, the use of NIV in plwALS varies from 15 to 21%.^46,49^ This underutilization of ventilatory support was potentially explained by patient choice, influenced by poor tolerance, reduced access, insufficient referral pathways or counterbalancing of benefits (extended lifespan, symptom alleviation) and burden (noise disturbance, dependence on medical equipment and nursing).

In the present study, the types of costs analysed are relatively similar to those considered in the open-source Data Pathologies platform.^50^ The annual expenditure for the overall management of plwALS in France (17 887€/PY) is higher than other neurological and degenerative diseases, such as multiple sclerosis, paraplegia and myasthenia (on average 10 809€, 10 093€ and 5577€ per person in 2021, respectively).^50^ In our study, most of the costs escalated at later stage, thus highlighting the importance of identifying and treating plwALS with early-stage ALS to prevent progression into a more resource-intensive stage. Higher costs at later stage correlate with other studies in ALS, including one from the USA where plwALS were compared to non-ALS matched controls.^51^

This study has several limitations that should be noted. The ICD code G12.2 for MND include ALS (accounts for >90% MND) and other MND diagnoses. Although the algorithm applied herein was refined after sensitivity analysis and efforts were made to specifically identify plwALS, it is possible that there were some instances of other motor neuron diagnoses included in the results. A 2016 study aimed to assess the accuracy of the French hospital discharge database and the health insurance data concerning the use of diagnosis codes for ALS case identification. The combination of the two data source yielded a high sensitivity and modest positive predictive value [93.8% (95% CI, 90.6–96.2); 60.8% (95% CI, 56.3–65.1)], respectively). Nevertheless, the authors concluded that diagnosis codes alone were not sufficient alone for case identification and that the use of supplementary data such as riluzole delivery is mandatory.^52^ According to the 2005 Consensus conference guidelines for the care of people with ALS, riluzole is introduced as soon as a diagnosis of ALS is suspected.^41^ A population-based study from the French ALS registry in Limousin revealed that 85.7% of ALS cases received a riluzole prescription.^53^ The algorithm used in our study was adapted from a 2017 nationwide study conducted by Kab *et al*. using the SNDS database. The estimates showed good consistency with data from the French Limousin ALS registry in terms of reported incidence rates.^3^ Second, as with any claims database, the SNDS data rely on the quality of information fed by HCPs and are subject to misclassification bias due to inaccurate diagnosis coding. Third, considering disease stages, SNDS does not capture clinical data such as ALSFRS-R which would allow for classification of King’s or MiToS ALS clinical staging. We employed a proxy symptom- and milestone-based algorithm, but this algorithm has not been validated in other studies and development was limited only to ALS survey data and physician input.^6^ Fourth, due to the constant evolution of the SNDS database, the data related to the cause of death managed by Inserm-CepiDc (Centre d'épidémiologie sur les causes médicales de Décès) were available only up to 2017. Additionally, for out-patient consultations (ACE), there is some uncertainty around the quality of coding for physician’ specialties in SNDS, which is set by default as general medicine, when in reality it took place with neurologist or any other specialty, with no possibility of distinguishing them. Overall, the utilization of out-patient care is likely not comprehensively described due to multidisciplinary visits not being captured in SNDS. Furthermore, the cost analysis in this study included all healthcare expenditure reimbursed to plwALS, direct medical costs and certain direct non-medical costs. Daily allowances paid in the context of sick leave are available in the database, but only for the general scheme, excluding local mutualist sections, and were analysed only for 6254 assumed active working age plwALS.^20^ Information is limited to sick leaves eligible for compensation from the national health insurance, which typically range from 50 to 66.6% of the employee’s average salary, and does not include coverage from private insurers or other payers.^20^ Other direct non-medical costs such as home or automobile modification for improved mobility, indirect costs by loss of employment and earnings, and any financial impact on the caregiver, and other out-of-pocket expenses were not captured. Thakore *et al*.^54^ reported direct costs to be remarkably lower than indirect costs and considered workspace absenteeism as a recurring indirect cost. Therefore, the cost estimates in this study are likely to underestimate the real cost of illness. Although patients with long-term chronic diseases, including MND, are reimbursed for all health expenditures, the actual utilization of healthcare services may vary due to disparities in access to care or socio-economic status.^42^ The scarcity of specialized HCPs or inadequate referral pathways may result in the underutilization of certain healthcare services among plwALS. Distance to care centres may also affect the overall cost and quality of care as ALS management typically requires the involvement of multidisciplinary teams centralized in specialized centres. Further research could explore the impact of these factors in the management of ALS in France. Lastly, while statistically significant differences were observed across subgroups, the large sample size may have introduced a greater probability of Type II errors. No mathematical correction was made for multiple comparisons.

Nonetheless, our study has several strengths. This is the first longitudinal study focusing on the natural history of ALS and capturing related HCRU through the comprehensive coverage of over 66 million persons, almost 99% of the French population,^19^ and thus provides representative insights of the population that would be difficult to ascertain from other clinical sources. The staging algorithm employed herein was built under the supervision of the SSC and was refined through multiple rounds of sensitivity analysis. We captured formal healthcare reimbursements with very low attrition rates, as reimbursements can be tracked even if the patient changes health insurer. The real-world nature and exhaustivity of the data, including specialized home care and assistive care, some of which are generally unavailable in the literature or patient registries, provide an insight into the real-life experiences of plwALS.

There is a need to improve diagnosis and treatment of ALS for the benefit of the patient and the healthcare system, as utilization/direct costs are lower in earlier stages when the patient has the fewest functional limitations. This observation confirms the crucial importance of understanding the mechanisms underlying motor neuron degeneration. It is also essential to promote the development of innovative neuroprotective therapies both within the scientific community and civil society. Our findings will support the infrastructure warranted to evolve new policies to safely detect, treat and prevent this disease. Herein, we emphasize the essentials of a strong support system for patients and their family through early genetic testing, improved early diagnosis and interventions and integrated multidisciplinary care and advocate for insurance coverage and policies that cover a wide spectrum of innovative therapies, palliative care and assistive and remote monitoring technologies to track ALS progression. Together, these measures will help alleviate the social and economic burden of ALS, thereby improving the quality of life of patients and caregivers while reducing the strain on the healthcare system.

## Conclusion

We demonstrate through a large national cohort of people that ALS imposes a public health burden through substantial incremental HCRU and costs, especially as the disease progresses. As the disease progresses, a decrease in the consumption of out-patient and ambulatory resources was seen in plwALS in France, accompanied by a corresponding increase in in-patient healthcare use whether in short-term institutions, aftercare and rehabilitation, or home care. Our findings give an insight into the changing needs of plwALS and can serve as a basis for development of innovative and integrative management strategies for this patient population. Such strategies may help reduce the economic expenditures and socio-economic burden as well as improve patient outcomes through early diagnosis and treatment.

## Supplementary Material

fcaf292_Supplementary_Data

## Data Availability

Data from the French administrative healthcare databases (SNDS) were available on the SNDS portal. Codes used for the ALS severity staging algorithm are available in Supplementary Table 1B.
