# Supplementary material for: Epidemiology, disease evolution and economic burden of amyotrophic lateral sclerosis in France using the French national health data system
Source: Brain Commun. 2025 Aug 11;7(4):fcaf292. doi: 10.1093/braincomms/fcaf292 (PMC12368419; doi:10.1093/braincomms/fcaf292)
Supplement: fcaf292_Supplementary_Data [file fcaf292_supplementary_data.pdf]

Supplementary Materials

Supplementary Table 1. A: Definitions of the three stages of ALS

Early	Middle	Late
Initiation of oral form riluzole<br>First MND diagnosis<br>First consultation with Neurologist<br>Electromyography<br>Measurement of conduction velocities<br>Signs and symptoms (Cramps and spasms, Fasciculations, Unspecified abnormal involuntary movements, Abnormalities of gait and mobility, lack of coordination, Abnormal reflex, Malaise and fatigue, Dysarthria, Mild malnutrition)<br>AND not having the events defining mid- and late-stage ALS	Initiation of liquid form riluzole<br>Initiation of anti-cholinergic drugs<br>Initiation of spasticity drugs<br>Reimbursement of walking sticks, wheeled walkers and manual<br>Reimbursement of positioning splints/ orthoses<br>Signs and symptoms (Dysphagia, Disturbances of salivary secretion, Dyspnea, Moderate to severe malnutrition)<br>Enteral nutrition use, Placement of gastrostomy<br>Non-invasive ventilation < 12 hours<br>Pneumonia<br>AND not having the events defining late-stage ALS	Communication aids devices<br>Electric wheelchair<br>Parenteral nutrition use<br>Non-invasive ventilation > 12 hours<br>Invasive ventilation<br>Tracheal suction<br>Tracheostomy<br>Respiratory failure<br>Admission to palliative care<br>Death

Supplementary Table 1. B: ALS Codes for Each Severity Stage

**Early-stage ALS**	
**Symptom or milestone**	**Code**
Initiation of oral form riluzole	CIP[a] codes: 34009 393 495 0 3, 34009 393 496 7 1, 34009 393 497 3 2, 34009 393 499 6 1, 34009 341 646 8 2, 34009 393 487 8 0, 34009 393 488 4 1, 34009 393 489 0 2, 34009 393 490 9 1, 34009 393 491 5 2, 34009 393 492 1 3, 34009 393 493 8 1, 34009 393 494 4 2, 34009 346 269 8 2, 34009 346 270 6 4, 34009 346 271 2 5, 34009 346 272 9 3, 34009 346 273 5 4, 34009 346 274 1 5, 34009 346 275 8 3, 34009 346 276 4 4, 34009 499 125 2 0, 34009 578 979 4 9, 34009 578 980 2 1, 34009 578 981 9 9, 34009 578 982 5 0, 34009 578 983 1 1, 34009 578 984 8 9, 34009 578 985 4 0, 34009 578 986 0 1, 34009 578 987 7 9, 34009 276 471 8 5, 34009 499 126 9 8, 34009 276 472 4 6, 34009 499 127 5 9, 34009 499 128 1 0, 34009 499 129 8 8, 34009 499 131 2 1, 34009 499 133 5 0, 34009 499 134 1 1, 34009 578 978 8 8, 34009 220 087 8 3, 34009 220 088 4 4, 34009 220 089 0 5, 34009 581 693 0 4, 34009 220 090 9 4, 34009 581 694 7 2, 34009 581 695 3 3, 34009 273 709 3 9, 34009 399 854 2 8, 34009 399 855 9 6, 34009 399 856 5 7, 34009 399 857 1 8, 34009 399 858 8 6, 34009 399 859 4 7, 34009 399 860 2 9, 34009 399 861 9 7, 34009 218 389 0 9, 34009 218 390 9 8, 34009 218 391 5 9, 34009 218 392 1 0, 34009 218 393 8 8, 34009 581 211 6 6, 34009 581 212 2 7, 34009 217 768 8 1, 34009 217 769 4 2, 34009 217 770 2 4, 34009 217 771 9 2, 34009 581 018 1 6, 34009 301 648 2 2, 34009 416 072 3 3, 34009 499 101 6 8, 34009 499 124 6 9, 34009 224 250 0 9, 34009 495 000 5 5
First MND diagnosis	ICD-10[b] code: G12.2
Electromyography	CCAM[c] codes: AHQB001, AHQB006, AHQB013, AHQB015, AHQB024, AHQB025, AHQB026, AHQB027, AHQB032, AHQB033
Measurement of conduction velocities	CCAM codes: AHQP003, AHQP008, AHQP010, AHQP011, AHQP012
Signs and symptoms (Cramps and spasms, Fasciculations, Unspecified abnormal involuntary movements, Abnormalities of gait and mobility, lack of coordination, Abnormal reflex, Malaise and fatigue, Dysarthria, Mild malnutrition)	ICD-10 codes<br>R252 Cramp and spasm<br>R253 Fasciculation<br>R259 Unspecified abnormal involuntary movements<br>R262 Difficulty in walking, not elsewhere classified<br>R268 Other and unspecified abnormalities of gait and mobility<br>R292 Abnormal reflex<br>R53 Malaise and fatigue<br>R471 Dysarthria and anarthria<br>R478 Other and unspecified speech disturbances<br>E44.1 Mild protein-energy malnutrition
**Mid-stage ALS**	
Initiation of liquid form riluzole	CIP codes: 34009 275 660 1 1, 34009 300 731 7 9, 34009 300 731 8 6
Initiation of anti-cholinergic drugs (scopolamine (patch), amitriptyline (oral), atropine (drops))	CIP codes: 3400932849437, 3400957584863, 3400949003433, 3400949004478, 3400930351574, 3400930351635, 3400930573020, 3400955531005, 3400930573259, 3400930573198, 3400930572948, 3400930853795, 3400930853856, 3400930853627, 3400932275953, 3400932276264, 3400930650462, 3400933645472, 3400930650233, 3400933645533, 3400930650172, 3400932276325
Initiation of spasticity drugs (Botulinum toxin type A injections, Thiocolchicoside,	GHM[d] code : 01K04J<br>CCAM codes : PCLB002, PCLB003

Methocarbamol, Tetrazepam, Muscle relaxants, directly acting agents, Baclofene, Dantrolene, Chlormezanone, Gallamine)	CIP codes: 3400930250419, 3400930250587, 3400930632192, 3400930695357, 3400931108290, 400931108351, 3400931716181, 3400932156009, 3400932156177, 3400932240883, 3400932288212, 3400932725557, 3400932888238, 3400933246600, 3400933398743, 3400933398804, 3400933401108, 3400933401566, 3400933401795, 3400933529178, 3400933765279, 3400933809195, 3400933819361, 3400934392689, 3400934584039, 3400934601798, 3400934646393, 3400934646454, 3400934874390, 3400935248732, 3400935331458, 3400935331519, 3400935331687, 3400935344465, 3400935451484, 3400935503565, 3400935503626, 3400935503794, 3400935503855, 3400935606105, 3400935730381, 3400935730671, 3400935769183, 3400935882646, 3400935882707, 3400935970916, 3400935971166, 3400935971395, 3400935971456, 3400935971685, 3400935971746, 3400935971975, 3400935972057, 3400935972286, 3400935972347, 3400935972408, 3400935973818, 3400936025677, 3400936096066, 3400936106505, 3400936139732, 3400936139961, 3400936143005, 3400936164345, 3400936164406, 3400936164635, 3400936164864, 3400936171619, 3400936172449, 3400936196681, 3400936219328, 3400936471856, 3400936617650, 3400936617711, 3400936738348, 3400936738409, 3400936743199, 3400936748743, 3400936819863, 3400937546102, 3400938151169, 3400938151220<br>UCD [e] codes: 9021506, 9021512, 9055356, 9060624, 9096958, 9096964, 9054322, 9023988, 9023971, 9002816, 9002822, 9035218, 9094190, 9165056, 9154644, 9154650, 9165062, 9165079, 9155017, 9096958, 9187827, 9186561, 9203785, 3458403, 9203093, 9204744, 9204750, 9165056, 9216960, 9218143, 9219160, 9223345, 9224385, 9222073, 9225887, 9248598, 9223552, 9238217, 9228940, 9308998, 9234662, 9234679, 9228940, 9234679, 9241538, 9241768, 9241751, 9241745, 9246501, 9241774, 9203785, 9242532, 9224385, 9270907, 9248606, 9245507, 9340944, 9295764, 9242532, 9246501, 9251175, 35586,17244, 9265881, 9266886, 9269778, 9270244, 9269100, 20046, 9306143
Reimbursement of walking sticks, wheeled walkers and manual wheelchair	Walking sticks – LPP [f] codes: 1200764, 1270463, 1296787, 6210046, 6210052, 6210715, 6222552, 6222581, 6229695, 6230089, 6230110, 6233627, 6233952, 6236092, 6238091, 6242968, 6248178, 6255706, 6256114, 6256746, 6257237, 6257295, 6259176, 6259213, 6259242, 6272857, 6272863, 6273390, 6273414, 6273791, 6273845, 6277122, 6277926, 6279003, 6280118, 6281715, 6282637, 6284205, 6284300, 6284346, 6284524, 6284530, 6284636, 6284642, 6284760<br>Walkers: 1225646, 1260418, 1285619, 1290968, 6210135, 6210141, 6210158, 6222598, 6230103, 6234041, 6256108, 6257266, 6259236, 6261517, 6264409, 6264869, 6266986, 6269720, 6272840, 6273383, 6273851, 6281721, 6282531, 6284458, 6284613<br>Manual wheelchair - LPP codes: 4101353, 4101956, 4106296, 4107723, 4113391, 4118193, 4119643, 4122473, 4122600, 4123627, 4124680, 4128441, 4134364, 4142530, 4156928, 4164566, 4169670, 4179540, 4181576, 4183434, 4183859, 4184899, 4192278, 4194159, 4195615, 4222803, 4263950, 4278212, 4300348, 4302152, 4307824, 4324739, 4325302, 4327382, 4329040, 4342654, 4359293, 4371408, 4371555, 4375116, 4375613
Reimbursement of positioning splints/ orthoses	LPP codes: 2159526, 2107972, 2104525, 2104637, 2109534, 2113174, 2120790, 2122138, 2122776, 2124338, 2128365, 2139943, 2144980, 2145984, 2149440, 2152211, 2152837, 2154032, 2156864, 2161530, 2164622, 2165194, 2174460, 2179500, 2187439, 2190393, 2192630, 2199968, 7110393, 7110418, 7110453, 7110482, 7110588, 7112110, 7112601, 7113517, 7113523, 7114110, 7114132, 7114244, 7114267, 7114296, 7114385, 7114391, 7115663, 7115775, 7118874, 7118897, 7118905, 7121764, 7121770, 7121818, 7122114, 7127985, 7128335, 7129889, 7129910, 7129926, 7130668, 7130674, 7130852, 7130869, 7132644, 7132673, 7132710, 7132727, 7132756, 7132762, 7132785, 7132800, 7132816, 7133371, 7133388, 7133419, 7133425, 7133431, 7133454, 7133460, 7136547, 7136576, 7136582, 7141382, 7141502,7141560, 7141608, 7141933, 7142393, 142884, 7142890, 7142909, 7142921, 7143027, 7143837, 7143843, 7144920, 7144966, 7144972, 7144995, 7145003, 7145026, 7145049, 7145055,7148770, 7148898, 7148912, 7148929, 7149076, 7153847, 7155645, 7157041, 7157578, 7157667, 7157680, 7157779, 7158610, 7160333, 7160511, 7160534, 7160735, 7160770, 7160787, 7160913, 7160920, 7160936, 7161232, 7161835, 7168180, 7168263, 7169340, 7171302, 7172276, 7173577, 7173940, 7175837, 7175895, 7175903, 7178391, 7178830, 7178899, 7178907, 7178920, 7178942, 7178959, 7179019, 7179060, 7179077, 7179120, 7179143, 7179150, 7179195, 7179203, 7179249, 7179255, 7179261, 7179290, 7179309, 7179315, 7179321, 7179338, 7179344, 7179373, 7179380, 7179396, 7179410, 7179462, 7179551, 7180442, 7180459, 7181170, 7181186, 7181192, 7181200, 7181252, 7181281, 7181482, 7181536, 7181542, 7181559, 7181565, 7181571, 7181588, 7181648, 7181750, 7181772, 7181803, 7181849, 7181855, 7181878, 7181890, 7181909, 7181921, 7182145, 7182180, 7182205, 7182257, 7182263, 7182292, 7182317, 7182487, 7182493, 7182501, 7182518
Signs and symptoms (Dysphagia, Disturbances of salivary secretion, Dyspnea, Moderate to severe malnutrition)	ICD-10 codes<br>R13 Dysphagia<br>J690 Pneumonitis due to inhalation of food and vomit<br>K117 Disturbances of salivary secretion<br>R060 Dyspnea<br>E44.1 Mild protein-energy malnutrition<br>E43 Unspecified severe protein-energy malnutrition<br>E46 Unspecified protein-energy malnutrition<br>Z713 Surveillance et conseils diététiques<br>R63.4 Abnormal weight loss
Enteral nutrition use, Placement of gastrostomy	Enteral nutrition use - LPP codes: 1134323, 1115372, 1116325, 1117974, 1118241, 1118962, 1119631, 1103140, 1103417, 1104339, 1105497, 1108142, 1108320, 1109673, 1109957, 1111902, 1112103, 1112793, 1136730, 1137439, 1138440, 1140482, 1144920, 1146071, 1136256, 1163709, 1163804, 1164264, 1165683, 1165708, 1168411, 1169149, 1169729, 1114119, 1150380, 1150523, 1150871, 1150931, 1152019, 1152769, 1153102, 1153480, 1154099, 1121600, 1100413, 1124690, 1125270, 1125695, 1127180, 1128630, 1129517, 1129724, 1129747, 1130578, 1132502, 1181506, 1183965, 1184054, 1195075, 1195916, 1155800, 1156678, 1156980, 1157399, 1191002, 1192007, 1193780, 1174073, 1174676, 1175049, 1175262, 1176876, 1170224, 1171270, 1172140, 1173330, 1173688,

	1179923, 1180091, 1196809, 1197223, 1197275, 1197542, 1198091, 1198317, 1199080, 1199788, 1184427, 1185415, 1186403, 1187220, 1131827<br>Placement of gastrostomy - CCAM codes: HFAD001, HFCA002, HFCB001, HFCC002, HFCH001, HFKD001, HFKE001, HFKH001, HFSA001<br>ICD-10 codes: Z43.1, Z931<br>GHM codes : 06M17Z, 06C231, 06C232, 06C233, 06C234, 06C23J, 06M17T
Non-invasive ventilation < 12 hours	LPP codes: 1100229, 1103720, 1107579, 1119134, 1125790, 1126708, 1130236, 1132442, 1158329, 1162437, 1169304, 1172967, 1196270, 1105184, 1112050, 1123414, 1126401, 1132270, 1138953, 1145723, 1163951, 1176480, 1188885, 1190161<br>CCAM codes: GLLD002, GLLD019, GLLD003
Pneumonia	ICD-10 codes: J12, J13, J14, J15, J16, J17, J18<br>GHM codes : 04M053, 04M052, 04M05T, 04M051, 04M054
**Late-stage ALS**	
Communication aids devices	LPP codes : 2443894, 2454024, 2470081, 7443166, 7448206, 7478093<br>CCAM codes : GKGD002, GKGE002, GKKD002, GKKE002, HEME002, HESA010
Electric wheelchair	LPP codes : 4113920, 4101821, 4108190, 4111854, 4116165, 4122250, 4122757, 4124331, 4130136, 4147668, 4152847, 4159170, 4168966, 4169492, 4307994, 4308597, 4309674, 4313760, 4321630, 4339681, 4342654, 4348622, 4379114, 4389845, 4390162, 4233570, 4252810
Parenteral nutrition use	CCAM codes : HSLF002, HSLF003<br>LPP codes : 1141487, 1145410, 1120522, 1100850, 1130354, 1155963, 1192510, 1185680<br>CIP codes: 3400931942009, 3400921787719, 3400922122267, 3400922122328, 3400922122557, 3400922122618, 3400922122786, 3400930076545, 3400930076552, 3400930077429, 3400930077436, 3400930077450, 3400930077467, 3400930077474, 3400935188908, 3400935189097, 3400935189158, 3400935189967, 3400935190048, 3400935428714, 3400935603371, 3400935603432, 3400935603661, 3400935635600, 3400935635778, 3400935635839, 3400935636089, 3400936987746, 3400936987807, 3400936988057, 3400936988118, 3400936988286, 3400937231534, 3400937231763, 3400937231824, 3400937231992, 3400937232074, 3400937232135, 3400937774567, 3400937774796, 3400937774918, 3400937775229, 3400937775519, 3400937775748, 3400938287394, 3400938287455, 3400938287684, 3400938287806, 3400938288285, 3400938288346, 3400938660593, 3400938660654, 3400938660715, 3400938661316, 3400938661484, 3400938661545, 3400939280790, 3400939280912, 3400939281223, 3400949821938, 3400949822010, 3400949822188, 3400949823420, 3400949823598<br>UCD codes: 0000009093931, 0000009384189, 0000009235868, 0000000040412, 0000009235845, 0000000040422, 0000000040417, 0000000048007, 0000000048008, 0000000047815, 0000000047816, 0000000048009, 0000009196737, 0000009196743, 0000009190798, 0000009196803, 0000009196826, 0000009182764, 0000009228029, 0000009228035, 0000009228041, 0000009235868, 0000009235839, 0000009235845, 0000009235779, 0000009292642, 0000009292659, 0000009292671, 0000009292688, 0000009292694, 0000009177237, 0000009177243, 0000009177266, 0000009177272, 0000009177289, 0000009177295, 0000009317112, 0000009317129, 0000009317135, 0000009317075, 0000009317081, 0000009317098, 0000009309845, 0000009309851, 0000009309816, 0000009309822, 0000009309874, 0000009309880, 0000009318057, 0000009318063, 0000009318086, 0000009318123, 0000009318146, 0000009318152, 0000009331307, 0000009331276, 0000009331282, 0000009368204, 0000009368173, 0000009368196, 0000009368144, 0000009368150
Non-invasive ventilation > 12 hours	LPP codes: 1100614, 1107763, 1108917, 1122053, 1125287, 1127607, 1144103, 1145404, 1151190, 1163030, 1166820, 1175380, 1177663, 1178540<br>CCAM codes : GLLD012
Invasive ventilation	LPP codes: 1101950, 1107042, 1116880, 1119826, 1144468, 1146444, 1150635, 1167570, 1167890, 1173560, 1182612, 1186544, 1192119, 1195520, 1199558<br>CCAM codes: GLLD004, GLLD006, GLLD007, GLLD008, GLLD009, GLLD011, GLLD013, GLLD015
Tracheal suction	LPP codes : 1149514, 1102375, 1106485<br>CCAM code : GEJE003
Tracheostomy	CCAM codes :GELF001, GEPA004<br>LPP codes : 1121421, 1133690, 1143470, 1165938, 1167937<br>ICD-10 codes: Z43.0, J95.0, Z93.0
Respiratory failure	ICD-10 codes: Z99, Z991, J9610, J96
Admission to palliative care	GHM codes: 23Z02T, 23Z02Z, 23Z03Z<br>ICD-10 code : Z51.5

[a] CIP= Code Identifiant de Présentation; [b] ICD-10= International Classification of Diseases, Tenth Revision; [c] CCAM= Classification Commune des Actes Médicaux; [d] GHM= Groupes Homogènes de Malades; [e] UCD= Unité Commune de Dispensation; [f] LPP= Liste des Produits et Prestations (LPP)

Supplementary Table 2: Top 20 most common medical causes of deaths among ALS patients in France from 2012 to 2017

Medical causes of deaths [a]	2012	2013	2014	2015	2016	2017
**N**	716	1,497	1,600	1,659	1,654	1,678
R09.2 Respiratory arrest	198 (27.65%)	409 (27.32%)	407 (25.44%)	460 (27.73%)	480 (29.02%)	433 (25.8%)
J96.0 Acute respiratory failure	171 (23.88%)	311 (20.77%)	330 (20.63%)	375 (22.6%)	356 (21.52%)	343 (20.44%)
J96.9 Respiratory failure, unspecified	67 (9.36%)	172 (11.49%)	176 (11%)	131 (7.9%)	146 (8.83%)	137 (8.16%)
R99 Other unspecified causes of mortality	52 (7.26%)	104 (6.95%)	86 (5.38%)	104 (6.27%)	106 (6.41%)	107 (6.38%)
J18.9 Pneumonia, unspecified	44 (6.15%)	101 (6.75%)	90 (5.63%)	103 (6.21%)	96 (5.8%)	84 (5.01%)
J69.0 Pneumonitis due to food and vomit	33 (4.6%)	110 (7.35%)	103 (6.44%)	148 (8.92%)	133 (8.04%)	136 (8.1%)
J96.1 Chronic respiratory failure	41 (5.73%)	60 (4.01%)	70 (4.38%)	89 (5.36%)	80 (4.84%)	69 (4.11%)
I46.9 Cardiac arrest, unspecified	39 (5.45%)	63 (4.21%)	75 (4.69%)	67 (4.04%)	68 (4.11%)	68 (4.05%)
R13 Dysphagia	22 (3.07%)	47 (3.14%)	68 (4.25%)	63 (3.8%)	77 (4.66%)	69 (4.11%)
I10 Essential (primary) hypertension	25 (3.49%)	69 (4.61%)	65 (4.06%)	44 (2.65%)	67 (4.05%)	66 (3.93%)
R26.3 Immobility	19 (2.65%)	50 (3.34%)	47 (2.94%)	60 (3.62%)	49 (2.96%)	57 (3.4%)
R09.0 Asphyxia	25 (3.49%)	40 (2.67%)	41 (2.56%)	53 (3.19%)	54 (3.26%)	49 (2.92%)
E46 Unspecified protein-energy malnutrition	20 (2.79%)	30 (2%)	47 (2.94%)	53 (3.19%)	52 (3.14%)	42 (2.5%)
R53 Malaise and fatigue	17 (2.37%)	32 (2.14%)	47 (2.94%)	47 (2.83%)	43 (2.6%)	44 (2.62%)
Z515 Palliative care	10 (1.4%)	30 (2%)	38 (2.38%)	43 (2.59%)	47 (2.84%)	57 (3.4%)
R402 Coma, unspecified	13 (1.82%)	35 (2.34%)	29 (1.81%)	46 (2.77%)	40 (2.42%)	35 (2.09%)
W79 Inhalation and ingestion of food causing obstruction of respiratory tract	8 (1.12%)	18 (1.2%)	29 (1.81%)	47 (2.83%)	41 (2.48%)	36 (2.15%)
R68.8 Other specified general symptoms and signs	18 (2.51%)	36 (2.4%)	25 (1.56%)	28 (1.69%)	30 (1.81%)	35 (2.09%)
R64 Cachexia	18 (2.51%)	23 (1.54%)	33 (2.06%)	29 (1.75%)	33 (2%)	33 (1.97%)
Respiratory causes [b]	468 (65.36%)	944 (63.06%)	973 (60.81%)	1,030 (62.09%)	1,049 (63.42%)	987 (58.82%)

[a]One patient can have multiple medical causes of death recorded in CepiDC database

[b]Respiratory causes include R09.2 Respiratory arrest, J96.0 Acute respiratory failure, J96.9 Respiratory failure, unspecified, J18.9 Pneumonia, unspecified, J69.0 Pneumonitis due to food and vomit, J96.1 Chronic respiratory failure, R09.0 Asphyxia, and W79 Inhalation and ingestion of food causing obstruction of respiratory tract

Supplementary Table 3: Treatment characteristics of ALS patients in France

		Number of patients, n (%)
**Patients treated with riluzole, n (%)**		
	Yes	13,455 (73.57%)
	No	4,834 (26.43%)
**Time from diagnosis to riluzole initiation (months)**		
	N	13,455
	Mean (SD)	0.66 (1.74)
	Median	0.07
	Q1-Q3	0-0.62
**Duration of riluzole treatment (months)**		
	N	13,455
	Mean (SD)	18.56 (18.12)
	Median	13.55
	Q1-Q3	5.73-24.43

Supplementary Figure 1: **A.** Outpatient consultation at disease severity stage; **B.** Outpatient consultation rate at disease severity stage

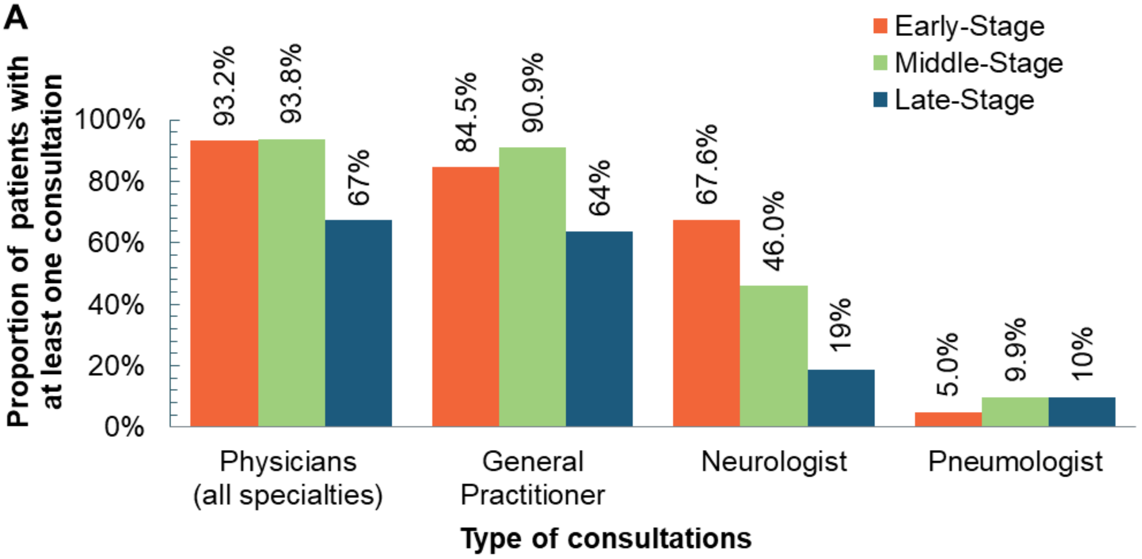

***N:** Early stage = 11,215; Mid stage = 15,098; Late stage = 16,025.*
***Statistical method:** Chi-squared test was used, and all observed differences were statistically significant ($p < 0.01$).*

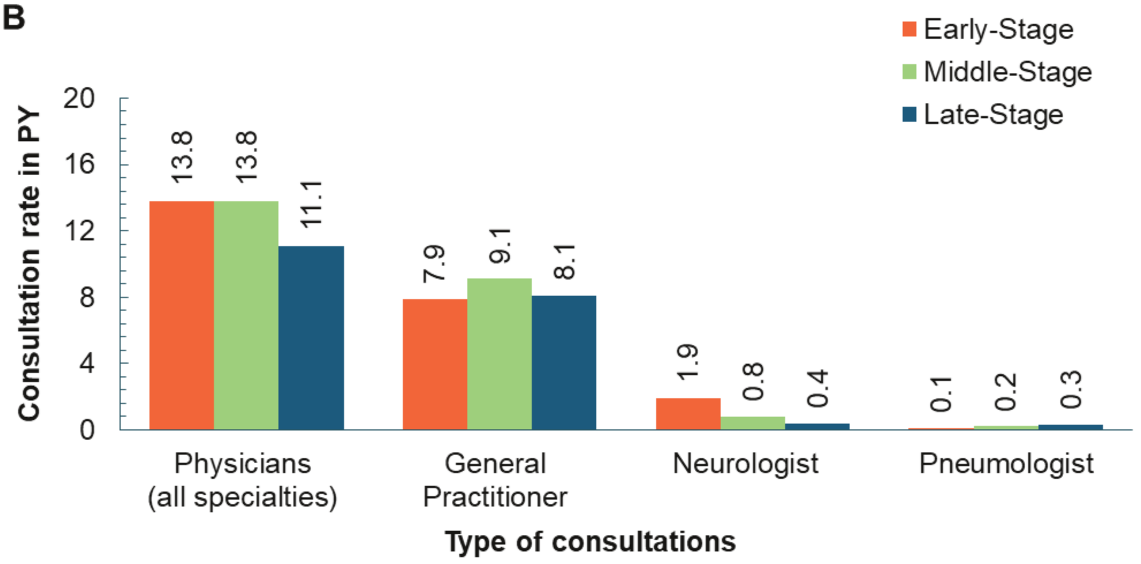

***N:** Early stage = 11,215; Mid stage = 15,098; Late stage = 16,025.*
***Statistical method:** Analysis of variance (ANOVA) was used, and all observed differences were statistically significant ($p < 0.01$). **Abbreviations:** PY (Person-Year).*

Supplementary Figure 2: A. Outpatient consultation for ALS vs non-ALS population; **B.** Outpatient consultation rates in patient years for ALS vs non-ALS population.

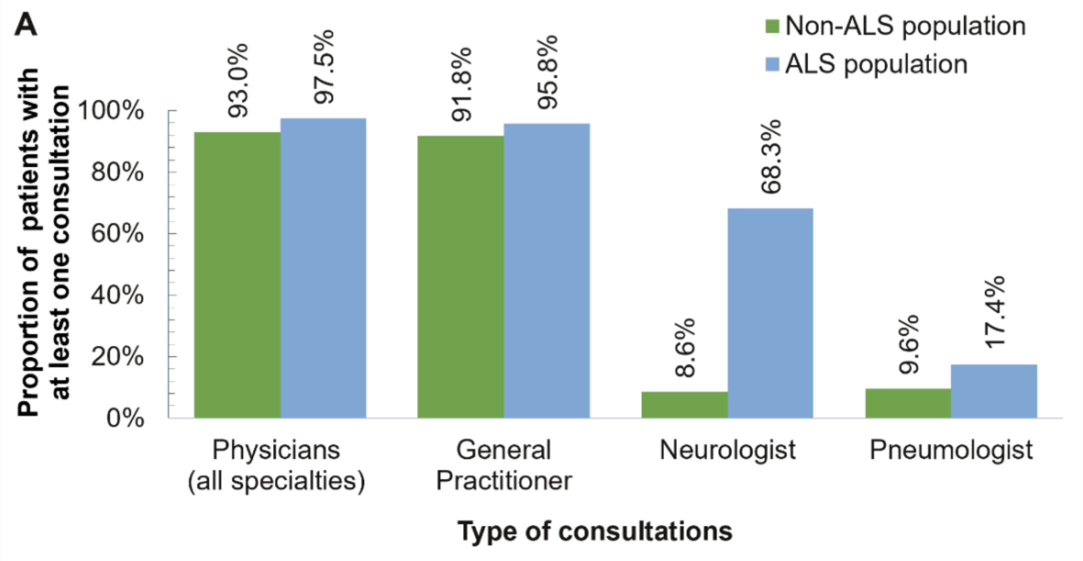

***N:** Non-ALS population=54,867; ALS population=18,289.*
***Statistical method:** Chi-squared test was used, and all observed differences were statistically significant (p < 0.01).*

***N:** Non-ALS population=54,867; ALS population=18,289.*
***Statistical method:** Student t-test was used, and all observed differences were statistically significant (p < 0.01). **Abbreviations:** PY (Person-Year).*
